# Urinary [TIMP-2] × [IGFBP7] and serum procalcitonin to predict and assess the risk for short-term outcomes in septic and non-septic critically ill patients

**DOI:** 10.1186/s13613-020-00665-9

**Published:** 2020-04-21

**Authors:** Ilaria Godi, Silvia De Rosa, Francesca Martino, Simona Bazzano, Marina Martin, Elisa Boni, Maria Rosa Carta, Claudia Tamayo Diaz, Gaia Mari, Anna Lorenzin, Massimo de Cal, Valentina Corradi, Carlotta Caprara, Davide Giavarina, Claudio Ronco

**Affiliations:** 1 International Renal Research Institute of Vicenza (IRRIV), San Bortolo Hospital, Vicenza, Italy; 2 Department of Medicine–DIMED, Section of Anesthesiology and Intensive Care Medicine, University of Padova, Padua, Italy; 3 Department of Anesthesiology and Intensive Care Medicine, San Bortolo Hospital, Vicenza, Italy; 4 Department of Nephrology, Dialysis and Transplantation, San Bortolo Hospital, Vicenza, Italy; 5 Department of Anesthesiology and Intensive Care, Azienda Ospedalieriero-Universitaria Maggiore DELLA Carità, Novara, Italy; 6 Department of Laboratory Medicine, San Bortolo Hospital, Vicenza, Italy

**Keywords:** Acute kidney injury, Sepsis, Intensive care unit, [TIMP-2] × [IGFBP7], Procalcitonin

## Abstract

**Background:**

Biomarkers can play a critical role by facilitating diagnosis and stratification of disease, as well as assessment or prediction of disease severity. Urinary tissue inhibitor of metalloproteinase-2 and insulin-like growth factor binding protein 7 product ([TIMP-2] × [IGFBP7]) predict the development and progression of AKI and recently procalcitonin (PCT), a widely used biomarker for sepsis diagnosis and management, has been associated with AKI occurrence in ICU patients. To assess combinations of [TIMP-2] × [IGFBP7] and PCT results for prediction and risk stratification of short-term outcomes in septic and non-septic patients, a retrospective cohort analysis of critically ill patients was performed in a multidisciplinary ICU. ROC curve analysis was used in order to evaluate predictive performance of combined results of [TIMP-2] × [IGFBP7] and PCT at the time of admission for AKI development. To verify the utility of adding [TIMP-2] × [IGFBP7] and PCT results for risk assessment, we evaluated the predictive value of having a single-marker positivity compared to a double-marker positivity using the widely used cut-off of 0.3 (ng/mL)^2^/1000 for [TIMP-2] × [IGFBP7] and 0.5 μg/L for PCT. Risk assessment for AKI occurrence within 48 h, acute kidney disease (AKD) and mortality at 7 days was performed by logistic/Cox regression analysis.

**Results:**

433 patients were analysed, of whom 168 had AKI within 48 h (93 septic and 65 non-septic patients). Combination of [TIMP-2] × [IGFBP7] and PCT showed a good predictive ability for AKI occurrence (AUC 0.81, 95% CI 0.77–0.86, *p* < 0.001, Sens 78%, Spec 73%). Combinations of biomarkers increased the odd ratios (OR) considerably. A single-marker positivity showed a fourfold risk increase, while the double-marker positivity a 26-fold risk increase for AKI occurrence. Moreover, the double-marker positivity showed an elevated risk for AKD at 7 days in non-septic patients (OR 15.9, 95% CI 3,21–73,57, *p* < 0.001) and for mortality within 7 days in septic patients (HR 4.1, 95% CI 1.4–11.8, *p* = 0.001).

**Conclusions:**

Although combining the results of [TIMP-2] × [IGFBP7] and PCT may be a useful tool to identify and stratify ICU patients at high risk for septic AKI and short-term adverse outcomes, data should be confirmed in a large prospective study.

## Introduction

Acute kidney Injury (AKI) occurs in 1 to 66% of hospitalized patients, depending on the economic status [1]. It is a frequent complication of serious acute illness and sepsis is one of the common causes [2]. AKI is associated with increased morbidity, mortality and high health costs [3, 4].

In this context, biomarkers may play a critical role by facilitating diagnosis and stratification of disease, as well as assessment or prediction of disease severity or response to therapy. Among the most recent ones, urinary tissue inhibitor of metalloproteinases-2 (TIMP-2) and insulin-like growth factor binding protein 7 (IGFBP7) product have been proven to be a good predictor of AKI [5] and received FDA approval for AKI risk assessment in critically ill patients. Recently, procalcitonin (PCT), a widely used biomarker for sepsis diagnosis and management, showed a significant association with AKI development [6]. Despite this association, the usefulness of adding PCT levels to existing models for predicting the occurrence of AKI in critically ill patients is still not clear [7]. Nevertheless, a combination of kidney-specific and non-kidney-specific biomarkers may contribute to early identification of AKI and stratification of critically ill patients. Here, we conducted this study to evaluate the relationship between combinations of [TIMP-2] × [IGFBP7] and PCT results and AKI in critically ill patients. Our hypothesis is that the admission [TIMP-2] × [IGFBP7]/PCT status may help to assess the risk for adverse renal outcomes in septic and non-septic populations.

## Methods

### Study population

We conducted a retrospective cohort study of adult patients consecutively admitted to a multidisciplinary ICU from June 2016 through February 2018. Critically ill patients were included in the study if they had [TIMP-2] × [IGFBP7] and PCT measurements on ICU admission. We excluded patients who were in the ICU for less than 24 h or did not have sufficient clinical information available.

The study was approved by local Ethics Committee of the San Bortolo Hospital (Vicenza), with protocol number of 03/17, and conformed to the Declaration of Helsinki. Informed consent was obtained under Italian laws.

### Definitions

Baseline serum creatinine (SCr) level was defined as the most recent SCr during 3–6 months before admission when available. In case of lacking data, the baseline SCr was considered admission SCr [8]. Back-estimation formula was not considered because in patients with suspected CKD, it overestimates the incidence of AKI [9].

AKI was defined and staged according to KDIGO 2012 consensus guidelines as follows: stage 1 as an increase in SCr level by ≤ 0.3 mg/dL), or an increase in SCr level 1.5–1.9 times the baseline value or urine volume < 0.5 mL/kg/h for 6–12 h; stage 2 as an increase in SCr level 2.0–2.9 times the baseline value or urine volume < 0.5 mL/kg/h for ≥ 12 h; stage 3 as an increase in SCr level 3.0 times baseline or ≥ 4.0 mg/dL, or initiation of renal replacement therapy (RRT) [10].

AKD was defined as a condition in which AKI stage 1 or greater was present 7 days after an AKI initiating event [11], excluding patients who died or were discharged from the hospital within the observation period. AKD staging was defined as follows: stage 0 as a SCr level < 1.5 times baseline but not back to baseline levels; stage 1 as a SCr level 1.5–1.9 times baseline; stage 2 as SCr level 2.0–2.9 times baseline; stage 3 as SCr level 3.0 times baseline or ongoing need for RRT.

Sepsis was defined according to Sepsis-III consensus definition as an acute change in total Sequential Organ Failure Assessment (SOFA) score ≥ 2 points subsequent to an infection [12]. The baseline SOFA score was assumed to be zero in patients not known to have pre-existing organ dysfunction. Infection status was defined as present in the subsequent conditions: (1) critically ill patients with confirmed infection (infective cause as reason of ICU admission); (2) critically ill patients with suspected infection in those who received empirical antibiotics after collections of body fluids for microbiological sampling within 24 h [13].

[TIMP-2] × [IGFBP7] value > 0.3 (ng/mL)^2^/1000 was considered positive while a value ≤ 0.3 negative [14].

A PCT value > 0.5 μg/L was considered positive while a value ≤ 0.5 negative.

### Measurements and data collection

Blood and urine samples were collected immediately after enrollment and analysed to measure serum creatinine and urinary [TIMP-2] × [IGFBP7] concentrations, respectively. Blood samples were also collected from day 1 to day 7 of ICU stay in order to measure SCr concentrations.

Serum creatinine was measured using the enzymatic method with an automatic analyser (Dimension Vista, Siemens Healthcare, Tarrytown, NY). Urinary [TIMP-2] × [IGFBP7] was analysed using Astute 140 m (Astute Medical), which divides the concentrations of the two biomarkers by 1000 to report a single numerical value in units of (ng/mL)^2^/1000.

Quantitative analysis of procalcitonin was performed using BRAHMS PCT sensitive KRYPTOR (ThermoFisher Scientific, Hennigsdorf, Germany). All laboratory tests were performed blinded to the clinical data.

For all patients, investigators collected from electronic health records baseline demographics, including age, sex, body mass index, comorbidities (hypertension and diabetes mellitus), reason for ICU admission, RRT requirement, duration of ICU care, duration of hospital care, duration of mechanical ventilation and vasopressors need laboratory findings and markers of clinical status (lactates levels, mean arterial pressure, diuresis). The glomerular filtration rate was estimated using the MDRD formula (eGFR) on admission. Severity of illness was calculated using the SOFA score and Simplified Acute Physiology score 2 (SAPS 2) to predict ICU mortality.

### Statistical analyses

The primary endpoint was to evaluate the predictive ability of AKI development within 48 h for results of [TIMP-2] × [IGFBP7] and PCT, alone and combined. Secondary endpoint was to assess the utility of combining the two biomarkers results for risk assessment of AKI within 48 h, AKD at 7 days and mortality within 7 days. We also examined primary and secondary endpoints in septic and non-septic subgroups.

Continuous data were analysed using Mann–Whitney tests for non-normal data. Categorical data were analysed using Pearson Chi-square test as appropriate.

A receiver operating characteristic curve (ROC) was created for [TIMP-2] × [IGFBP7], PCT and the combination of the two biomarkers plotting the probabilities from regression model [15]. Using Youden’s test, we determined an optimal cut-off and analysed its sensitivity and specificity. We also evaluated [TIMP-2] × [IGFBP7] with a validated cut-off of 0.3 and PCT with a clinical cut-off of 0.5, analysing their sensitivity, specificity, positive and negative predictive values. Logistic regression analysis was performed to assess odds ratio (OR) of AKI occurrence within 48 h and results were adjusted for variables resulted significant in backward stepwise likelihood ratio model.

In order to evaluate the utility of adding [TIMP-2] × [IGFBP7] and PCT results, we assess the risk for the single-biomarker positivity (when [TIMP-2] × [IGFBP7] or PCT resulted positive) and the double-biomarker positivity (when both biomarkers tested positive) relative to the negativity of both biomarkers.

All results yielding a *p* value less than 0.05 were determined to be statistically significant.

Data were analysed using SPSS version 22.0 (IBM Corporation, Armonk, NY).

## Results

### Patients characteristics

**Fig. 1:**
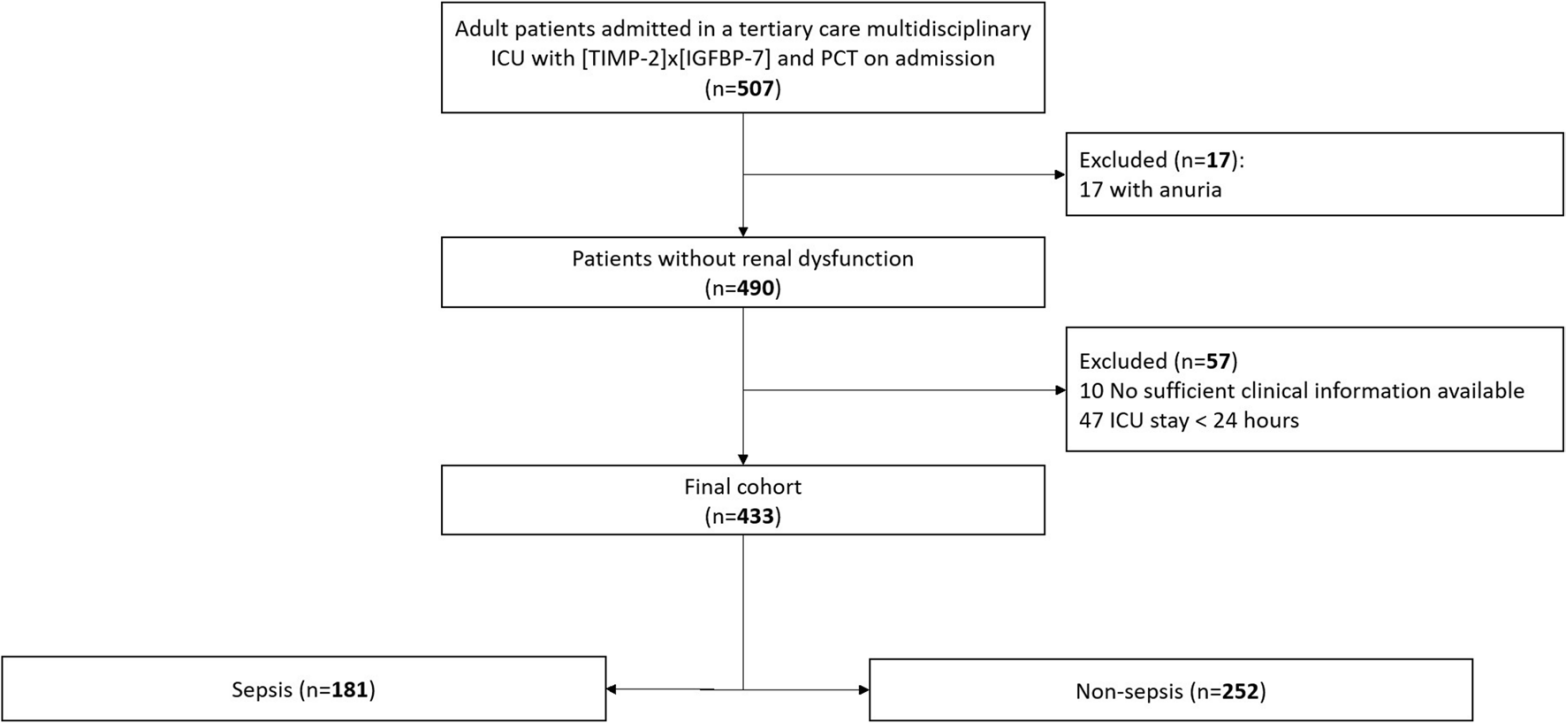
Flowchart of study design. The chart shows patient inclusion and exclusion in the study analysis. ICU: intensive care unit; [TIMP-2] × [IGFBP7]: urinary tissue inhibitor of metalloproteinases-2 and insulin-like growth factor binding protein 7 product

Flowchart of the study design is shown in Fig. 1. During the study period, 507 adult ICU patients had [TIMP-2] × [IGFBP7] and PCT on admission. One hundred seventy-one patients were excluded: 17 with anuria, 10 with no data available and 47 with ICU stay less than 24 h.

Among 433 patients eligible for analysis, 181 were included in septic and 252 in non-septic cohorts. A documented baseline creatinine was present in 288 patients and admission creatinine was used as reference creatinine in 145 patients. Basal characteristics are described in Table 1.

**Table 1:** Demographic data and admission characteristics

	Analysis cohort	No AKI	AKI	*p*-value
Patients	433	265	168	
Age (years)	70 (56–78)	69 (54–78)	72 (60–80)	0.042*
Male gender	272 (62.8)	164 (61.9)	108 (64.3)	0.018*
BMI	25 (23–29)	25 (23–28)	26 (23–31)	< 0.001*
Admission reason				0.001*
Infection	85 (19.6)	35 (13.2)	50 (29.8)	
Trauma	70 (16.2)	50 (18.9)	20 (11.9)	
Postoperative care	68 (15.7)	41 (15.5)	27 (16.1)	
Neurologic	61 (14.1)	51 (19.2)	10 (6.0)	
Cardiovascular	55 (12.7)	35 (13.2)	20 (11.9)	
Respiratory	48 (11.1)	29 (10.9)	19 (11.3)	
Others	46 (10.6)	24 (9.1)	22 (13.1)	
Comorbidities				
Hypertension	226 (52.2)	126 (47.5)	100 (59.5)	0.015*
Chronic kidney disease	53 (12.2)	8 (3.0)	45 (26.8)	< 0.001*
Diabetes mellitus	80 (18.5)	39 (14.7)	41 (24.4)	0.011*
SOFA score	7 (5–10)	6 (4–9)	8 (6–11)	< 0.001*
SAPS2 score	50 (36–63)	38 (27–50)	42 (27–53)	0.375
Invasive ventilation time (days)	4 (2–10)	3 (1–10)	4 (2–9)	0.038*
Vasopressor time (days)	1 (0–1)	1 (0–1)	1 (0–2)	< 0.001*
eGFR (mL/min/1.73 m^2^)	81 (59–99)	89 (70–104)	68 (43–89)	< 0.001*
Mean arterial pressure (mmHg)	79 (68–90)	80 (70–104)	73 (65–86)	< 0.001*
Lactates (mmol/L)	2.0 (1.4–3.5)	1.7 (1.2–2.6)	2.6 (1.7–5.1)	< 0.001*
Baseline creatinine (mg/dL)	0.83 (0.66–1.08)	0.77 (0.62–0.95)	0.98 (0.77–1.35)	< 0.001*
Urinary [TIMP-2] × [IGFBP7] (ng/mL)^2/1000	0.5 (0.1–2.1)	0.22 (0.07–0.99)	1.39 (0.47–5.72)	< 0.001*
Procalcitonin (mg/L)	0.5 (0.1–4.6)	0.2 (0.1–0.9)	3.2 (0.6–17.6)	< 0.001*

Demographic data and admission characteristics. Data are reported as numbers (percentages) as categorical variables and median (interquartile range) for continuous variables. * identified a *p*-value < 0.05

*BMI* body mass index, *SOFA* Sequential Organ Failure Assessment, *SAPS2* Simplified Acute Physiology score 2, *eGFR* estimated glomerular filtration rate, *[TIMP-2] × [IGFBP7]* tissue inhibitor metalloproteinase 2 and insulin-growth factor binding protein 7 product

### [TIMP-2] × [IGFBP7] and PCT levels at the time of admission and AKI development

Of the 433 patients, 168 (38.8%) developed AKI within 48 h, of which 55 (12.7%) were categorized as stage 1, 44 (10.2%) as stage 2 and 69 (15.9%) as stage 3 (Table 2). Patients with AKI at ICU admission were 6.0% (*n* = 26), 28.4% (*n* = 123) had AKI between admission and 24 h, and 4.4% (*n* = 19) had AKI between 24 and 48 h.

**Table 2:** Patients’ outcomes in the overall population and in septic and non-septic subgroups

	Analysis cohort	Sepsis	Non-sepsis	*p*-value
Patients	433	181	252	
AKI at ICU admission	26 (6.0)	14 (7.7)	12 (4.8)	0.001*
Stage 1	14 (3.2)	8 (4.4)	6 (2.4)	
Stage 2	5 (1.1)	2 (1.1)	3 (1.2)	
Stage 3	7 (1.6)	4 (2.2)	3 (1.2)	
AKI within 24 h	149 (34.4)	82 (45.3)	67 (26.6)	< 0.001*
Stage 1	46 (10.6)	25 (13.8)	21 (8.3)	
Stage 2	38 (8.8)	19 (10.5)	19 (7.5)	
Stage 3	65 (15.0)	38 (21.0)	27 (10.7)	
AKI within 48 h	168 (38.8)	93 (51.4)	75 (29.8)	< 0.001*
Stage 1	55 (12.7)	31 (17.1)	24 (9.5)	
Stage 2	44 (10.2)	22 (12.2)	22 (8.7)	
Stage 3	69 (15.9)	40 (22.1)	29 (11.5)	
RRT need	33 (7.6)	18 (9.9)	15 (6.0)	
AKD at 7 days	47 (10.8)	26 (14.4)	21 (8.3)	< 0.01*
Stage 0	15 (3.5)	10 (5.5)	5 (2.0)	
Stage 1	9 (2.1)	5 (2.8)	4 (1.6)	
Stage 2	7 (1.6)	4 (2.2)	3 (1.2)	
Stage 3	16 (3.7)	7 (3.9)	9 (3.6)	
RRT need	14 (3.2)	7 (3.9)	7 (2.8)	
7 days mortality	65 (15.0)	37 (20.4)	28 (11.1)	< 0.01*
ICU mortality	100 (23.1)	50 (27.6)	50 (19.8)	0.57
Days in ICU	4 (2-11)	4 (2–10)	4 (2–11)	0.77
Hospital mortality	128 (29.6)	64 (35.4)	64 (25.4)	0.37
Days in hospital	15 (7–31)	13 (6–30)	15 (7–31)	0.27

Data are reported as numbers (percentages) as categorical variables and median (interquartile range) for continuous variables. * identified a *p*-value < 0.05

*AKI* acute kidney injury, *AKD* acute kidney disease, *RRT* renal replacement therapy, *ICU* intensive care unit

Patients with AKI within 48 h were older (72 vs 69 years, *p* = 0.042), with more acute critical illness (SOFA score 8 vs 6, *p* < 0.001), with a prevalence of infective cause for ICU admission. 59.5% of them had a history of hypertension, 24.4% diabetes mellitus and 26.8% chronic kidney disease. These patients also had lower eGFR (89 vs 68 mL/min/1.73 m^2^, *p* < 0.001) and mean arterial pressure (80 vs 73 mmHg, *p* < 0.001) on admission, higher lactate levels (1.7 vs 2.6 mmol/L, *p* < 0.001) and needed mechanical ventilation (3 vs 4 days, *p* = 0.038) and vasopressors for longer period (1 [0–1] vs 1 [0–2] days, *p* < 0.001). [TIMP-2] × [IGFBP7] values were higher in AKI patients compared to those who did not develop AKI (0.63 [0.13–2.19] vs 0.23 [0.06–0.59], *p* < 0.001), as well as PCT (1.20 [0.20–6.70] vs 0.20 [0.10–0.90], *p* < 0.001).

In order to evaluate the biomarkers predictive ability for AKI occurrence, we performed a ROC curve analysis. [TIMP-2] × [IGFBP7] showed a fair predictive ability for AKI development (AUC 0.75, Sens 83%, Spec 59%), as well as PCT (AUC 0.79, Sens 79%, Spec 67%). The combination of the two biomarkers slightly increased the predictive ability for AKI occurrence (AUC 0.81, 95% CI 0.77–0.86, *p* < 0.001, Sens 78%, Spec 73%). In the overall population, the optimal cut-offs of [TIMP-2] × [IGFBP7] were higher than the validated cut-off of 0.3 and showed higher specificity, while optimal cut-off of PCT approximated the clinical one. The results of ROC curve analysis (AUCs, optimal cut-offs, Sens and Spec) for the entire cohort and for subgroups are detailed in Additional file 1: Table S1 and Additional file 2: Table S2.

### AKI risk assessment by [TIMP-2] × [IGFBP7] and PCT results and their combination

Logistic regression analysis was performed to assess odds ratio (OR) of AKI occurrence within 48 h using the validated cut-off of 0.3 for [TIMP-2] × [IGFBP7] and the widely used cut-off of 0.5 for PCT. After multivariate analysis which incorporated admission eGFR and SOFA score, the positivity of the biomarkers was associated with an increased risk for AKI occurrence (Table 3). Of note, PCT > 0.5 was not an independent predictor of AKI in septic population. To evaluate the utility of adding [TIMP-2] × [IGFBP7] and PCT results for risk assessment, we evaluated the predictive value of having a single-marker positivity compared to a double-marker positivity. As shown in Table 3, the presence of at least one of the two biomarkers was significantly associated with AKI development (OR 4.1, 95% CI 1.9–8.8, *p* < 0.001) and when both biomarkers were positive the probability of AKI occurrence strongly increased (OR 26.4, 95% CI 12.3–56.62, *p* < 0.001). If the results were confirmed in non-septic population, within septic patients only the positivity of both biomarkers significantly increased the risk for AKI occurrence with a 19-fold risk increase.

**Table 3:** Risk assessment for primary and secondary outcomes in the entire population and in septic and non-septic subgroups

Variables	Analysis cohort			Sepsis			Non-sepsis		
	OR	95% CI	*p*-value	OR	95% CI	*p*-value	OR	95% CI	*p*-value
Primary outcome									
AKI within 48 h									
[TIMP-2] × [IGFBP7] > 0.3	3.93^a^	2.14–7.20	< 0.001	5.92^a^	2.53–13.82	< 0.001	3.27^a^	1.53–6.97	< 0.001
PCT > 0.5	3.67^a^	2.17–6.19	< 0.001	2.74^a^	0.87–8.57	0.083	4.85^a^	2.37–9.94	< 0.001
Single-marker positivity	4.08	1.90–8.76	< 0.001	2.27	0.45–11.23	0.316	4.93	2.05–11.82	< 0.001
Double-marker positivity	26.41	12.32–56.62	< 0.001	19.5	4.20–90.44	< 0.001	25.11	9.58–65.79	< 0.001
Secondary outcomes									
AKD at 7 days									
Single-marker positivity	4.73	1.04–21.60	0.045	2.28	0.26–20.02	0.15	3.24	0.65–16.20	0.151
Double-marker positivity	15.92	3.67–68.97	0.001	4.57	0.48–36.25	0.15	15.36	3.21–73.57	0.001
Mortality within 7 days									
Single-marker positivity	1.16	0.51–2.65	0.724	0.86	0.77–2.75	0.494	1.59	0.63–4.05	0.329
Double-marker positivity	2.75	1.34–5.65	0.006	4.1	1.41–11.78	0.001	2.01	0.75–5.40	0.166

A single-marker positivity was defined by the presence of [TIMP-2] × [IGFBP7] above the cut-off of 0.3 or PCT above the cut-off of 0.5; the double-marker positivity was defined by the presence of [TIMP-2] × [IGFBP7] measurements above 0.3 and the concomitant presence of PCT levels above 0.5

*[TIMP-2] × [IGFBP7]* tissue inhibitor metalloproteinase-2 and insulin-growth factor binding protein 7 product, *PCT* procalcitonin

^a^ Odds ratios (OR) were adjusted for Sequential Organ Failure Assessment (SOFA) and estimated glomerular filtration rate (eGFR) at the time of admission

### Association of combined positivity of [TIMP-2] × [IGFBP7] and PCT with AKD at 7 days

To determine a potential association with persistence of AKI, a subset analyses was performed excluding patients who died or were discharged from the hospital within the observation period (*n* = 136). 47 of 297 patients had AKD at 7 days (15.8%), of whom 14 were classified in stage 0 (*n* = 26 in septic group), 9 in stage 1 (*n* = 5 in septic group), 7 in stage 2 (*n* = 4 in septic group), 16 in stage 3 (*n* = 7 in septic group) and 14 required RRT (*n* = 7 in septic group). As shown in Table 3, a double-biomarker positivity had a 10.3 risk increase for AKD at 7 days (*p* = 0.001). These results were confirmed in non-septic population, where the risk for AKD was 15.9 times higher when both biomarkers tested positive.

### Association of combined positivity of [TIMP-2] × [IGFBP7] and PCT with mortality within 7 days

Of the 433 patients analysed, 65 died within 7 days (*n* = 37 in septic group). Using Cox regression analysis, only the double-marker positivity showed an increased risk for mortality within 7 days (Table 3) and these results were confirmed only in septic patients (HR 4.1, 95% CI 1.4–11.8, *p* = 0.001).

## Discussion

AKI is a commonly encountered complication of critical illness, associated with significant mortality, long-term adverse outcomes and high costs [16]. AKI and its progression could be prevented or mitigated by effective care strategies if sufficient warning is provided [10]. Sepsis is one of the most common causes of AKI in critical care patients and the two syndromes can interweave in the condition of sepsis-associated AKI [17].

Biomarkers are potential tools that offer early warning where intervention can still alter outcomes. Some biomarkers revealed activity in biological pathways that precede AKI and other biomarkers are triggered by the presence of subclinical AKI. Therefore, combining the results of biomarkers involved in different steps of disease process may be helpful in the prediction and assessment of the risk.

The [TIMP-2] × [IGFBP7] product is known to be a biomarker of kidney stress in multiple settings [18], while PCT is recognized as a suitable marker for sepsis (or infection) diagnosis and has recently been associated with AKI (contrast-induced AKI [19] and pancreatitis-associated AKI [20] and critically ill patients [7]).

Some characteristics may link PCT and [TIMP-2] × [IGFBP7] in AKI and in septic AKI. First, both biomarkers can be upregulated by ROS in the context of hypoperfusion and hypoxia. ROS production have been shown to induce PCT expression [21] and to be a potential stress insult to induce TIMP-2 and IGFBP7 production [22].

Second, both biomarkers might be expression of an inflammatory status, more or less kidney-specific. In vitro results demonstrated that peripheral blood mononuclear cells (PBMCs), as well as endothelial cells, were potentially capable of contributing to in vivo extrathyroidal PCT production [23]. Activated PBMCs and pro-inflammatory cytokines, such as tumour necrosis factor alpha and interleukin 1 are considered to play a role in PCT production and inflammation [24]. In a recent study by Gocze et al., a tubular stress detected by [TIMP-2] × [IGFBP7] positivity was associated with a systemic γδ T cell immune cell response [25].

The main findings of this study were the subsequent: (1) the combination of the biomarkers demonstrated a good predictive ability for patients at risk for AKI occurrence; (2) combined results according to widely used cut-offs of 0.3 for [TIMP-2] × [IGFBP7] and 0.5 for PCT allowed risk stratification of the population for AKI development within 48 h; (3) A double-marker positivity, defined by the concomitant positivity of both biomarkers was also significantly associated with mortality within 7 days in septic subgroup and with AKD at 7 days in non-septic patients.

Several clinical studies have evaluated the utility of [TIMP-2] × [IGFBP7] product in the early diagnosis and risk stratification of AKI [5, 26]. Honore et al. found that [TIMP-2] × [IGFBP7] accurately predicted AKI in septic patients [27]. Our study found a predictive ability of [TIMP-2] × [IGFBP7] for AKI in critically ill and septic patients lower than previously described. Furthermore, the cut-off proposed by Hoste et al. [14] showed a good sensitivity, but a low negative predictive value. These findings probably reflected the heterogeneity of our ICU population as well as the study design. It should be taken also into account that the primary endpoint was development of any stage of AKI. Surprisingly, PCT showed a better predictive ability compared to cell cycle arrest biomarkers, both in septic and non-septic groups.

Literature proved PCT to be a strong marker of infection and sepsis and recently PCT have been significantly associated with subsequent AKI development in critically ill patients [7]. Despite sepsis, other noninfective conditions, such as surgery, trauma, burn, pancreatitis, renal dysfunction can also increase PCT levels [28]. In some of our patients, PCT was elevated regardless of their non-septic condition and the most common admission reason was trauma. Other admission causes were postoperative care, neurological, cardiovascular and respiratory causes. Even though the association between PCT and AKI exists, the usefulness of adding PCT levels to the existing models is still not clear for predicting the occurrence of AKI in critically ill patients in the intensive care unit (ICU) and the interpretation of PCT elevation on admission as signal of AKI requires caution.

Therefore, we investigated the combination of [TIMP-2] × [IGFBP7] and PCT for AKI prediction with the hypothesis that the addition of an AKI biomarker with a sepsis biomarker may lead to early identification of patients with sepsis-induced AKI in order to direct preventive or proactive strategies and preserve renal function. Our results demonstrate that combining biomarkers enabled enhanced risk prediction by way of a composite risk assessment. Furthermore, using the validated cut-off of 0.3 for [TIMP-2] × [IGFBP7] and the widely used cut-off of 0.5 for PCT, we can stratify patients according to the presence of single or double-biomarker positivity. The combined positivity of the two biomarkers had approximately 26-fold increase of AKI risk, which represents a very large increase in OR from 4.0 when only one of the two biomarkers was positive. Whether this increase might be considered clinically relevant will depend on the baseline risk of the patients. Interestingly, these results were confirmed in septic and non-septic patients. The findings that [TIMP-2] × [IGFBP7] and PCT combined increased the risk for AKI also in non-septic patients led to considering two possible hypotheses. Firstly, PCT a surrogate marker of sepsis; in this line, the combination of the two biomarkers may help in risk prediction and stratification for AKI and sepsis and may highlight [TIMP-2] × [IGFBP7] to be not only an AKI biomarker, but also a marker of sepsis. Previous trials studied PCT as a marker of sepsis after surgery and trauma, however evidence is lacking [29, 30]. A second hypothesis may be the association between inflammation and subsequent AKI development. In consideration of that our ICU policy does not provide a routine use of PCT and the measurements were requested according to clinicians’ judgement, therefore our population have to be considered at risk for infection and inflammation. Nonetheless, we cannot conclude for any of the two hypothesis, because other markers of inflammation or infection should be evaluated.

A recent study also investigated urinary cell cycle arrest biomarkers in conjunction with classical markers of AKI to improve risk stratification for short-term severe outcomes [31]. PCT was found to be correlated with mortality only when serial measurements were performed [32]. We found that the combined positivity of [TIMP-2] × [IGFBP7] and PCT was associated with an increased risk for mortality within 7 days in septic patients and for AKD at 7 days in non-septic patients. We also found that the combined positivity reflected a more critical pattern both in septic and non-septic groups. For instance, the positivity of both biomarkers was associated with increased risk of mortality within 7 days, possibly reflecting the most critically ill patients, especially those with confirmed or suspected sepsis. From the other side the double-marker positivity combination at the time of admission was associated with an increased risk for AKD at 7 days in non-septic patients, suggesting that a septic/inflammatory state increased the risk for persistence of the renal insult. This is the first study to explore the relationship between combination of urinary cell cycle arrest biomarkers and PCT and short-term outcomes in septic and non-septic critically ill patients.

Our study has several limitations. First, this is a retrospective analysis with a small sample size in a single centre. As a secondary analysis of data from a prospective observational study, available information is limited. We have no information on the focus of infection and pathogens detected. Second, the fact that PCT levels may be affected by either acute or chronic renal dysfunction is under debate [24, 33] and the diagnostic accuracy of PCT level may be lower in patients with AKI or CKD. On the other hand, selection bias should be taken into account because of the exclusion criteria used. Of note, for research purpose [TIMP-2] × [IGFBP7] was measured in all patients admitted to our ICU during the study period. The results were therefore adjusted for factors that were linked to the endpoints. Third, we performed only baseline measurements of biomarkers under investigation and cannot clarify the variability in biomarkers during the study, whilst ICU patient’s status may change dramatically within hours. Fourth, AKI within 24 and 48 h may be viewed as a debatable timeframe; indeed, some patients may develop AKI afterwards. In addition, AKD was handled as a non-time-dependent variable, whereas this variable is strongly affected by time dependency, even though we tried to limit this bias by excluding from analysis those patients who died or were discharged from the hospital.

Despite such limitations, the study might be relevant for clinical implication and further research on sepsis and AKI field.

## Conclusions

[TIMP-2] × [IGFBP7] and PCT can be used to identify critically ill patients at risk for AKI occurrence. The combination of [TIMP-2] × [IGFBP7] and PCT with cut-offs of 0.3 and 0.5, respectively, may help in stratifying the risk for AKI, regardless of sepsis. The present study also showed that the combined biomarkers elevation over the cut-off values was a predictor for mortality within 7 days in patients with suspected or confirmed sepsis and for AKD at 7 days within patients without sepsis.

Although combining the results of [TIMP-2] × [IGFBP7] and PCT could be in the future a useful tool for assessing the risk for septic AKI and short-term adverse outcomes, data should be confirmed in a large prospective study.

## Supplementary information

**Additional file 1.** ROC curve analysis for [TIMP-2] × [IGFBP7], procalcitonine and the combination of the two biomarkers.

**Additional file 2.** ROC curve analysis for [TIMP-2] × [IGFBP7] and procalcitonine with predefined cut-offs of 0.3 and 0.5 respectively, and the combination of the two results.

## Data Availability

All data analysed during the current study are available from the corresponding author on reasonable request.

## Abbreviations

AKI: Acute kidney injury
AKD: Acute kidney disease
CKD: Chronic kidney disease
HR: Hazard ratio
ICU: Intensive care unit
IQR: Interquartile range
OR: Odds ratio
PCT: Procalcitonin
SAPS2: Simplified Acute Physiology score 2
SCr: Serum creatinine
SOFA: Sequential Organ Failure Assessment
[TIMP-2] × [IGFBP7]: Urinary tissue inhibitor metalloproteinase-2 and insulin-like growth factor binding protein 7 product
